# 787. Predictors of Co-infection with Multidrug Resistant Organisms among Hospitalized Patients with COVID-19: An Exploratory Analysis

**DOI:** 10.1093/ofid/ofac492.048

**Published:** 2022-12-15

**Authors:** Yue Zhang, Haojia Li, Barbara E Jones, Michael Rubin, Candace Haroldsen, Tina M Willson, Atim Effiong, Jessica Cole, Karim Khader

**Affiliations:** School of Medicine, the University of Utah, Salt Lake City, Utah; University of Utah, Salt Lake City, Utah; University of Utah and Salt Lake City VA Healthcare System, Salt Lake City, Utah; University of Utah, Salt Lake City, Utah; University of Utah, Salt Lake City, Utah; University of Utah, Salt Lake City, Utah; University of Utah, Salt Lake City, Utah; University of Utah, Salt Lake City, Utah; University of Utah, Salt Lake City, Utah

## Abstract

**Background:**

Bacterial co-infection has been reported with COVID-19, but risk factors for bacterial co-infection remain unclear due to limited large scale studies. We seek to identify predictive factors associated with risk of co-infection with multidrug-resistant organisms for patients hospitalized at Veterans Affairs (VA) hospitals with COVID-19.

**Methods:**

This retrospective cohort study included Veterans admitted to VA hospitals from March 1, 2020 through May 31, 2022 with a confirmed positive COVID-19 test within the previous 14 days and up to 2 days after admission. Outcomes of interest were hospital-onset co-infection (HOI, > 2 calendar days after admission) and community-onset co-infection (COI, within 2 calendar days of admission). Potential risk factors included both patient- (e.g. vital sign, medication use) and facility-level covariates (e.g. bed size, antibiotic use rate). We compared the covariate distributions for patients with and without HOI and COI. Our analytical approaches included variance inflation factors to detect the presence of multicollinearity among these factors, and Least Absolute Shrinkage and Selection Operator to identify the subset of factors associated with HOI and COI. We conducted a two-stage analysis, first performing feature selection among the individual-level risk factors followed by identification of facility-level risk factors. Optimal models were identified using 10-fold cross validation.

**Results:**

By July 2021, 33,383 patients were admitted to VA with positive COVID-19 test. We found that medications for ventilator induction (OR with 95% CI: 2.9 (2.2, 3.9)), norepinephrine (OR with 95% CI: 1.6 (1.2, 2.2)) and antimicrobial therapies for gram-positive infections (OR with 95% CI: 4.5 (3.6, 5.6)) [Table 1] were associated with the increased risk of HOI and patients in facilities with high *C difficile* infection rates were more likely to have COI detected (OR with 95% CI: 1.14 (1.11, 1.18)) [Table 2]. Homeless Veterans had higher risk of developing an HOI (OR with 95% CI: 1.5 (1.1, 2.0)), but not a COI.

**Conclusion:**

Risk factors for HOI and COI in COVID-19 were distinct, with specific classes of medications and antibiotics as well as patient factors resulting in increased risk for HOI. Further work is needed to better understand the risk factors for COI.

**Disclosures:**

**Karim Khader, PhD**, BioFire Diagnostics: Grant/Research Support.

**Table 1:**
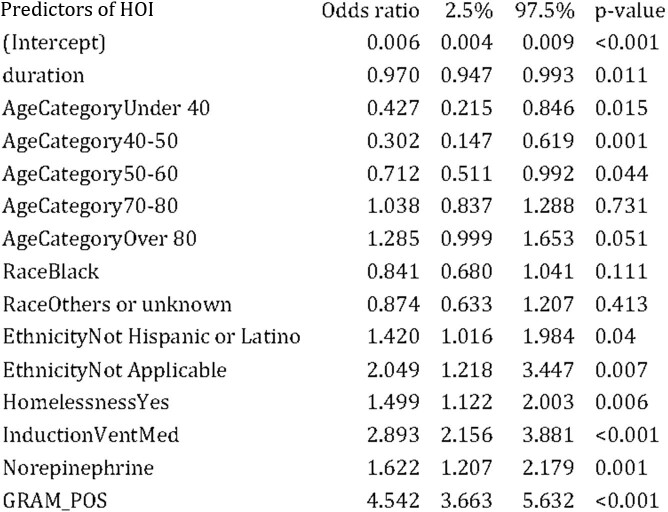
Mixed effects logistic regression on HOI (patient-level covariates only)

**Table 2:**
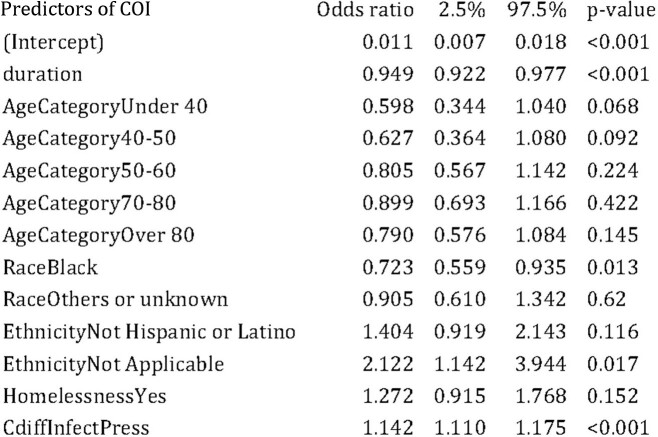
Mixed effects logistic regression on COI (patient-level and facility-level covariates)

